# Challenges of Researchers in Conducting International Study during the Eruption of COVID-19: Student and Mentor Perspectives

**DOI:** 10.3390/ijerph19020844

**Published:** 2022-01-12

**Authors:** Jenail Mobaraka, Lian Elkazzaz, Niveen Rizkalla

**Affiliations:** 1 Center for Middle Eastern Studies, University of California Berkeley, 340 Stephens Hall, Berkeley, CA 94720-2314, USA; jmobaraka@berkeley.edu; 2 Global Studies, University of California Berkeley, 101 Stephens Hall, Berkeley, CA 94720-2306, USA; lianelkazzaz@berkeley.edu

**Keywords:** global crisis, personal perspectives, qualitative research, refugees, aid workers, student-mentor relationship, Lebanon, COVID-19

## Abstract

Conducting an international research study may bear various challenges; however, during the global COVID-19 crisis, such a study undertakes unpredictable trajectories. This paper explores the challenges experienced by researchers studying Syrian refugees’ physical and mental health and aid workers serving under humanitarian organizations in Lebanon. It includes information about the changes in the study’s goals and design with the emergence and spread of SARS-CoV-2, as necessitated by the circumstances COVID-19 imposed. It focuses on the unique perspectives of the research team of two students and their mentor who faced multiple challenges while involved in the study, and their narratives and subjective experiences that led to new opportunities for growth in the project. The research team specifically engaged in humanistic and existential psychology in order to conduct research in a manner conducive to personal and professional development, productivity and growth. To conclude, the researchers propose recommendations to the academic community on mitigating some of the challenges faced when conducting international research, and suggestions to the humanitarian sector serving vulnerable populations in conflict zones during COVID-19.

## 1. Introduction

Since the onset of the COVID-19 pandemic, the international community has come to a breaking point. Borders have been closed, economies have suffered and communities have lived in fear of potential viral transmission. Academic research activities were significantly hampered, field visits and data collection were paralyzed and funding resources were frozen and halted [1]. In our study, the existing challenges of conducting international field work research in Lebanon’s precarious socio-economic and political conditions were compounded by the complex implications of COVID-19.

This paper explores the challenges encountered by the team of researchers from two perspectives: the students’ perspectives, the first two authors, and the mentor’s perspective, the third author. The paper begins with a brief background on the study and its complex circumstances and proceeds to the subjective perspectives of each researcher’s personal experience. The authors’ experiences will engage in a humanistic and existential psychology scholarship and its relevance to the process of this study.

In the initial study design, the research team approached contacts in the Middle East. It established connections with on-the-ground nongovernmental organizations (NGOs) operating in Lebanon, which provide services to Syrian refugees and local Lebanese in need. The study titled “Violent Conflict, Physical and Mental Health Needs of Syrian Refugees in Lebanon” aimed to examine the physical and mental health conditions and unmet needs of Syrian refugees who reside in the urban areas of Lebanon. The initial goals of the study did not include the impacts of COVID-19 on the targeted population. The students were set to travel to Lebanon to gather survey data from Syrian refugees and conduct semi-structured interviews with staff of the NGOs frequented by Syrian refugees for aid and services. However, due to COVID-19, travel-related restrictions were put into effect, which prevented the implementation of the initial study design, as well as changed the goals of the study. Various risks and ethical concerns, which arose around the continuation of the study, included the possibility of the students’ immediate deportation from Lebanon, their inability to return to the United States, and most importantly, the risk of transmitting the virus unknowingly to the already vulnerable refugee population and service providers who were assisting them. Moreover, the academic institution with which the team was affiliated has determined a strong preference for human subjects research to be limited, “unless it was in the best interest of the subjects” (R. Katz, personal communication, 16 March 2020). Thus, out of moral and ethical values, the study immediately shifted to be remote and focused on semi-structured interviews with only the staff of NGOs. The initial goals and design of the study to examine Syrian refugees’ physical and mental health needs—collected firsthand from refugees and NGO staff—were changed to examining Syrian refugees’ conditions as they were perceived by staff, as well as the physical and mental health consequences for staff due to their trauma work, while considering the impacts of COVID-19 on both refugees and staff [2,3].

During the COVID-19 pandemic, staff faced extreme limitations as a result of quarantine. This included the stress and mental health effects of deteriorating global conditions, changes in family dynamics and the enmeshment of boundaries, as well as complexities in the work–life balance, which shifted to a work-from-home life balance [4]. Staff had to rapidly adjust to their organizations’ new health and safety guidelines and faced increased workloads in serving communities who were struggling under shelter-in-place policies. As a collective society, social-distancing imposed difficulties on staff in restricting their social encounters, daily responsibilities, caregiving to family elderly/parents and their community support, which mostly includes frequent physical touch as a survival need, as well as a social and cultural norm for expressing human affection and warmth, especially common in the Middle East.

The shelter-in-place policies and the difficulties of conducting an international study during a pandemic was doubly challenging on researchers as well. Most participants were at home and therefore did not have access to the same work environment they did in their offices. This included a lack of private office space and the limited availability of a wireless connection; a condition in Lebanon where electricity inconsistencies are commonplace [5]. The limited internet connectivity forced researchers to only audio-record, rather than video-record aid workers, which had the benefits of minimizing a breach in participants’ confidentiality and enabled a safe space, but on the other hand, it narrowed the human interaction and nonverbal communication (e.g., body language, facial expressions) necessary for understanding emotional states. Furthermore, the 10-h time difference between Lebanon and California (Pacific Standard Time) posed additional stress. Establishing a rapport with participants in order to gather the data was paramount, but external distractions proved significant and required additional attention, flexibility and care [6,7].

Conducting an international study in the Middle East regardless of a pandemic is complex in itself. In terms of interviewer–interviewee dynamics, coming from a prestigious American academic institution to interview participants from the Middle East generates diverse power imbalances [8]. Participants were accustomed to interacting with American and other Western institutions in more formal donor–recipient relationships, rather than being interviewed about their personal working conditions and mental health, in addition to the physical and mental health of refugees and the locals they served. Moreover, the difference in cultural norms between participants and interviewers required adjustment for a cohesive and comfortable interview experience [9,10]. Ultimately, the dynamics of research conducted in the Middle East—where colonialism dominated the past, but still has many impacts on the reality in the present—by American researchers were only made more complex by the complications of living through a global pandemic and performing remote interviews.

All the aforementioned challenges have particular implications in the complicated socio-economic and political Lebanese terrain. Since October of 2019, Lebanon has undergone significant political upheavals, with millions of protestors taking to the streets to voice their frustration with governmental austerity and rapidly declining economic conditions [11,12]. Further, no one could have predicted the onset of the COVID-19 pandemic and its disastrous implications on the already vulnerable Lebanese state’s economy. The consequences were so grave that protests only took a brief hiatus with the imposition of government shelter-in-place orders, but reignited due to economic unrest.

Syrian refugees, the primary population we had interest in studying, have been severely impacted by both the economic–political tensions and the COVID-19 conditions in Lebanon. To some degree, everyone in the world has felt the invasion of this crisis in their personal, professional, financial and familial lives [13]. For refugees who have already lived through the traumatic events of war, horror journeys of escape and who were barely surviving the extreme post-displacement poverty and survival challenges in the host country [14,15,16,17,18,19,20], their sense of insecurity and shattered safety have been further fragmented and ruptured. COVID-19 has created an inhumane hostage situation for refugees recuperating from the wounds and the ramifications of war. The refugee experience is one of forced displacement—crossing borders to flee the violence within their homelands—in search of shelter, protection and freedom. However, with a borderless global crisis like COVID-19 looming, safety remains out of reach and exacerbates refugees’ predicament. Thus, COVID-19 was evidenced as a complex, multilayered, prolonged global and shared trauma, which has an adverse and disproportionate impact on Middle Eastern minorities [21]. Its impact on minorities, among which are Syrian refugees, was found to elevate PTSD symptoms, depression and anxiety due to prolonged economic and lockdown acute stressors, discrimination, as well as multiple ongoing traumatizations and their cumulative and proliferation dynamics [21,22].

## 2. Student Perspective: The First Author

As a senior in college, this project was my first opportunity to engage in research so thoroughly. It is not often that an undergraduate student gets the level of access that I did into scholarship of this magnitude. Despite this being an incredible opportunity, there also existed a personal need and pressure to complete this project. Conducting original research and writing an accompanying undergraduate thesis is a requirement for graduation and was meant to be the culmination of four years of arduous and thoughtful study in this field.

With the news of shelter-in-place and subsequent travel restrictions, funding I had previously secured to support my field research was rescinded because of federal restrictions on travel. The inability to conduct the in-person interviews our team planned was particularly difficult for me as it could not be postponed and would push back my graduation until the project was completed. Coming from a background where I had little experience in conducting research and with my ability to graduate linked to the successful completion of this project, my stress increased significantly. However, my determination to continue the project did not solely lie in a fear of sunk costs or failure. The opportunity to conduct this study, under the circumstances of a global pandemic, encompassed a humanitarian purpose: finding new meaning in life and a valuable responsibility, a personal vocation and mission as a Syrian American researcher, in the hope to serve my people in delivering the voices of their suffering to the public eye. Eric Fromm’s [23] analysis of the human condition may explain the mixture of excitement and distress I felt upon embarking on such a journey; with the freedom to choose comes a tremendous responsibility, and into that burdening role of authentically and truly vocalizing my people’s torment, protrudes anxiety.

As a Syrian American, I expected discomfort in conducting research related to the Syrian refugee crisis. I understood that hearing the stories of war survivors would be deeply upsetting as I am a Syrian who felt close to the conflict, but I did my best to prepare. Nonetheless and despite my preparations, the inhumane conditions Syrian refugees endured during COVID-19, especially those described by aid workers, many of whom were Syrian refugees themselves, were the most inspiring and painful narratives to which I was exposed. I was saddened to discover that structural violence imposed on refugees during COVID-19 was even more dehumanizing of their already fragile conditions. The pandemic exacerbated their needs and limited their access to services, which resulted in keeping them physically isolated, socially demonized and consequently made the public panic from mortality threats more tangible [24,25]. Refugees were alienated and abandoned to cope with minimal governmental assistance, which left all the burden and responsibility on humanitarian organizations and their staff.

Aid workers had become overwhelmed due to the increased amount of support they had to provide to their beneficiaries in remote interactions, new project implementation and their own personal statuses as residents, or as Syrian refugees who worked in Lebanon. Many considered themselves in an emergency situation and had to take calls and complete tasks after work hours, in addition to dealing with their own disturbed routines and economic stressors. These circumstances imposed additional challenges in scheduling a convenient time for interviews or conducting the interviews in a space that provided privacy.

Further into the research process, the effects of interviewing, transcribing and analyzing the responses of aid workers became difficult. COVID-19 had burdened and slowed down all activities, which at times felt extremely heavy to me and made the transcribing process slower than I anticipated [6,26]. Mental and physical fatigue had started to show its impact with a feeling of emptiness looming over and slowing the process further.

In the struggle to defeat this exhaustion among our research team, we needed to remind ourselves “why are we doing this?” The sense of purpose and sacrifice aid workers shared with me has inspired me, especially that of Syrian refugees, who felt obligated and committed to help their people and vocalize their suffering. I made a choice and drew strength to persist for the sake of the same meaningful goal they held onto; we were united by a meaning we had to fulfill [27]. Nietzsche’s words of wisdom, cited by [27] helped in understanding their mission and my own: “He who has a why to live for can bear almost any how”. Meaning-making is an important tool for coping with physical and mental health through life challenges such as adversity, crisis and trauma [28]; however, it was limited, especially among aid workers [2,3,29], and I have only encountered a few academic studies demonstrating similar impacts on researchers.

Practically, in order to persist and overcome this challenge, our research team agreed to edit each other’s interviews after finalizing the initial transcriptions. Analysis was conducted independently, which proceeded with group analyses and discussions on the final codes. The team shared the progress of the work via a group file, wherein all finalized materials were added by each researcher. This mutual responsibility and transparency of working together as a group improved our motivation and productivity, since the progression of the study depended on each team member’s contribution. This process particularly enlightened my understanding on how resourceful we were as a team, the power of solidarity [30] and how fortunate we were in being able to live in a country that still enjoyed abundance under such circumstances, especially in comparison to our participants. This realization and sense of gratitude allowed us as a research team to establish authentic relationships among one another, which were interwoven with empowered feelings of love and belongingness.

The friendships we formed with each other served as solace for me when the community was scarce, scattered, or too overwhelmed to offer any support. Our relationships also served as motivation for working through the project, even when it was emotionally taxing. Our resiliency in the face of unimagined challenges gave me hope for the future and a purpose in learning from the stories of aid workers I interviewed. My encounters with participants were filled with compassionate human connections that were not hampered despite being virtual.

Conducting international research during this time has been filled with challenges I never expected to face as an undergraduate student. However, because of the adaptation in this study I developed my research skills while limiting the margin of error that may have occurred in the field as a first-time researcher. Having spent four years studying the Middle East and personally being invested in the outcomes of the Syrian conflict, this project was gratifying at the culmination of my college career. This study taught me a lot about researchers’ existential need of walking alongside participants with sensitivity, the shared humanity between the researchers and participants [26], and the essence of compassion.

## 3. Student Perspective: The Second Author

Islamic tradition offers a saying, translated roughly as, “don’t hate something; it could be good for you”. I often found myself returning to this verse through our research project and the unfolding of the COVID-19 crisis. In its early stages, COVID-19 struck with collective fear and panic and revealed existential anxieties [13].

As a research team, we closely watched every development to plan our next moves. We sought travel and research-related guidance from the university and tracked the closure of airports and imposition of increasing travel restrictions globally. Ultimately, several months of planning unraveled over the span of two weeks. In making the decision to indefinitely postpone our travel plans, my frustrations exceeded my capacity as a student-researcher with disrupted plans. My Lebanese heritage emerged at the forefront of my thinking and I was left devastated at the prospect of losing the opportunity to reconnect with my family in Lebanon and revisit my invisible home and collective roots for the foreseeable future.

At the time of this project, I was a third-year undergraduate and a new transfer student from a community college. Thus, I had minimal research experience and faced a substantial learning curve. Prior to undertaking this project, I had never heard of research scales, much less tailored an entire project according to my interests. I quickly found myself doing work I previously thought inaccessible to undergraduates; I learned to code and analyzed transcripts and aided in protocol writing of the Institutional Committee for the Protection of Human Subjects review board.

Theoretically, government-imposed lockdowns would work to our advantage, as participants were working from home and would likely have a greater capacity to participate. In practice, we found that in the early days of lockdown, the world was still adjusting to life at home and social distancing. For weeks, our entire lives had been consumed by the virus; it occupied our thoughts, conversations and wedged its way into our relationships.

This invasion of sorts was abundantly present in my interviews through the demeanor of my participants, their responses and their outlooks. My questions were often met with “before COVID-19 or now?” It became clearer with each interview that participants were not interested in talking about circumstances prior to the onset of COVID-19. While I set out to learn about Syrian refugees’ legal and economic status in Lebanon, participants were more concerned with the implications of the rapidly deteriorating economy due to lockdown measures on the already extremely vulnerable refugee population. Thus, interview questions were modified to address the new goals of the study in examining aid workers’ current needs, concerns, and subjective experiences during the pandemic [6]. Although social and economic disparities have always been observed in Lebanon, I was still devastated to witness how COVID-19 intensified them, to the extent of splitting Lebanon aggressively into local-Lebanese and nonlocal refugees. During the pandemic, refugees were vilified, othered, and became even further alienated and feared. As a result, they suffered from heightened hostility and discrimination [21]. Hostility and micro-aggression were present in our interviews, when Lebanese aid workers articulated their professional opinions and perspectives on refugees while trying to hide their personal derogatory criticism and stigmatized attitudes.

Despite the complexity of conducting remote interviews, I bonded with many of my participants over our shared difficulties with working from home, the mental toll of quarantine and the emotional weight of witnessing the world’s uncertain state. As different as our circumstances were, I identified with my interviewees who, like myself, were working from home in less-than-ideal conditions, often with our families in the next room. I frequently found myself in an apology tug-of-war with the person on the other line, each insisting our regret for our poor internet connection. During the interviews, I learned how to differentiate between the line dis-connectivity and the silence I needed to allow in the conversation as a space for participants’ reflection [6] and gradually internalized not to fear from such silence [31].

In my previous academic and personal experience, I have found that navigating the Lebanese socio-political arena was tantamount to navigating a minefield. As a Lebanese person in the diaspora, I have always been invested in understanding the material conditions of the most vulnerable populations in the country. The personal narratives of aid workers have illustrated to me the magnitude of the challenges they and refugees face in ways that my past observations as an outsider could not. The participants effectively took my hand and walked me through the complexities of their work and lives in the Lebanese context. They discussed the technicalities of aid-related work in the Lebanese field, but also opened themselves up to be intimate and vulnerable with a stranger. By the conclusion of the interview process, I had discovered far more than what we initially sought out. Participants shared in great detail the structures of the NGOs serving refugees and highlighted their competition for the resources necessary to provide relief. I also identified my own vulnerabilities and learned new self-care techniques, as well as coping with pain, stress and trauma. Aid workers taught me about finding meaning and growth in humanitarian work and the factors which drove them to commit their professional and often personal lives in the service of others, despite the ever-present risk of secondary trauma [3,32].

However, the most important lesson they taught me was on how fragile we are as humans when confronting conflicts and disasters such as a pandemic, but not inasmuch as how resilient we could be in the most unanticipated moments; as in Nietzsche’s [33] vastly quoted affirmation: “That which does not kill us makes us stronger”. One of the Syrian participants attested that her son was killed in the war and she was still mourning his loss. Nonetheless, she was devoted and determined to contribute to other refugees at work. Her testimony reverberated within me how the personal, professional and political could not be differentiated, especially under the most miserable conditions, which force a person to transcend one’s self. She lost her son, but she wasn’t lost, she didn’t lose her will to live and survive, she said “yes” to life despite her loss and embraced an abundant lifestyle in the service of others.

Writing and disseminating this manuscript were also complex and anxiety provoking, especially in that journal reviews were also slowed down due to the pandemic circumstances and the unique scope of this paper, which did not always yield a perfect fit to journals’ aims. However, reading about Frankl’s determination—while being in a concentration camp, he clung onto rewriting his destroyed manuscript, which gave him a purpose to live and forced him to rise above his suffering—left our team with a feeling of awe. We too could not bear the thought of not finalizing this work under less severe conditions. The lessons we learned about the human capacity to make the best of any given situation by creatively turning negative aspects into constructive ones, under all circumstances, were tremendous. Acknowledging how meaningful our work and the relationships we established, have inspired us to change our attitudes towards the challenges of dissemination and to transcend above the quarantine sense of emptiness into proactively writing the manuscript. This has even rendered new opportunities for resilience and growth [27]. From this research team emerged loving and life-long friendships and as Yang [30] (p. 558) articulated: “it takes a team to accomplish important things”. In the rigorous academic environment through which we came together, this is a rarity. The nature of our dynamic provided collaborative space and support, which I will always value as essential components of success in every research endeavor, project and human interaction.

## 4. Mentor Perspective: The Third Author

Prior to COVID-19, my main concern as a mentor was related to the possible adverse impacts of secondary traumatic stress [32] and vicarious traumatization [34] of the students due to their involvement in a study that encompasses exposure to traumatic narratives. The students conducting the study were not professionally trained for their encounter with Syrian refugees who endured multiple traumatic events and prolonged survival stressors [15,16,17,18]. Therefore, the student researchers were at risk of potential harm in experiencing refugee PTSD-similar symptoms due to their field work-related exposure [2,3,35,36]. I was also concerned that the students’ lack of experience in interviewing traumatized populations, may result in causing discomfort or re-traumatization of participants [37]. Therefore, prior to the planned travel to Lebanon, I provided the students with training on conducting such a study, interviewing participants, participants’ vulnerability and potential benefits, self-care techniques, as well as increasing their awareness of the risk to their well-being as a consequence of their empathetic involvement with refugees via weekly in-person supervision meetings [37,38].

However, with the eruption of COVID-19, my concern amplified to include the potential harm to students as they were in similar circumstances to the study’s participants. They not only empathized with participants by putting themselves in their shoes, but they also identified with the participants being quarantined at home. This unusual and complex situation placed my students in a position defined as “Shared Traumatic Reality” [39,40]. Both the students and participants lived and worked under quarantine. This stressful context was at times traumatizing. Personally, I was also under quarantine, maneuvering parenting and academic work and faced similar stressful challenges; however, I needed to remain functional to provide my students with remote support and practical solutions during the research process. The more COVID-19 was hovering like an existential threatening cloud over our heads, the more the awareness of death became tangible and the greater my urge intensified in clinging onto life, similarly to confronting death as described by Irvin Yalom’s book [41] “The Gift of Therapy”. Though Yalom describes a more general awareness of death, my awareness of death was contextualized by the particular experience of fear due to the COVID-19 circumstances. Choosing life and living for me were reflected in a zest of vitality, enhanced awareness of the study’s importance and rarity, and a hectic working pace. Therefore, my relationship with my students intensified and became significant to a degree that they have saved me from isolation, lack of creativity and the ever-changing sense of uselessness and degrading meaninglessness [27].

The sudden change in the global spread of the virus made the study’s initial implementation plans impossible. Despite both students’ extreme desire to travel, I had to consult with administrative officials and colleagues regarding the university’s restrictions on travel. It was a difficult decision I had to make, bearing in mind both the health and safety of the students and participants, as well as the threat of the students’ inability to return to the United States from Lebanon. After long discussions with the students and navigating their disappointment and frustration, we decided to adhere to the university’s code of conduct and new guidelines and adjusted the study according to the new requirements the IRB office placed regarding international projects. This decision resulted in compromising many of the initial research goals that would have provided a person-centered holistic perspective on refugees’ conditions from both refugees and aid workers [42]. Instead, we accepted the fact that data collected might provide only partial information gathered from only aid workers and according to their subjective points of view. This solution of dividing the research into phases, wherein the first phase was conducted remotely, provided some relief to students and allowed for an easier adjustment to the remote phase.

Afterwards, the study’s team started preparing for the changes ahead. First, I contacted all the collaborating NGOs and informed them of the changes pertaining to our inability to conduct the study physically in Lebanon and gained their approval to conduct remote interviews with their staff. The response from organizations was slow due to being overwhelmed with the changes occurring globally and locally and the new stress their organizations faced in shifting their communication and services provided to refugees and locals to a remote operation. Then, the research team adjusted the research goals and interview schedule, to include new questions pertaining to the impacts of COVID-19 on aid workers and their perspectives on the impacts on beneficiaries. The informed consent process was adjusted to contain remote interviewing, ethical considerations of recorded interviews, privacy and confidentiality [31].

During the preparations for the changes in the study, I had to adjust the training of the students to weekly remote meetings, in addition to including potential challenges related to remote interviews. These challenges included the clarity of interview recordings, scheduling interviews considering the time difference, internet dis-connectivity and flexibility required from the researchers. Flexibility in conducting the study required scheduling meetings according to participants’ convenience and language preferences—which have not always aligned with those of the researchers. Fluency and professional terms in Arabic were another concern that students needed my help in translating. Additionally, we always had to bear in mind that participants were at their homes with their families, children, accompanying background noises, and distractions. Furthermore, other challenges were raised pertaining to participants’ responses, which might be biased to solely focusing their discourse on the impacts of COVID-19, rather than a chronological comparison prior to the pandemic. Moreover, it was necessary to consider participants’ levels of stress and the general feelings of panic in the Middle East. All these research challenges as well as personal challenges increased the students’ anxiety and stress levels in conducting a study for the first time in their academic experience, as well as my own anxiety in mentoring them during these unpredicted circumstances.

**Figure 1:**
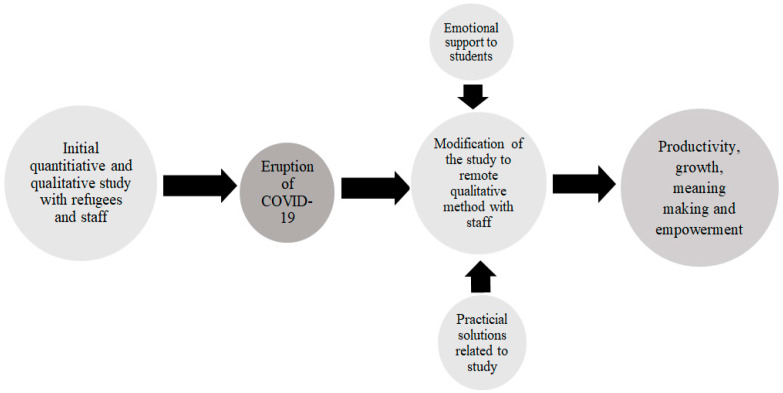
Challenges of researchers in conducting international study during the eruption of COVID-19.

As a Palestinian trauma researcher, it was important to me to support my students during these tumultuous times. Therefore, our weekly remote meetings would commence with the students sharing their personal experiences, state of mind and well-being and later on would proceed to the research-related challenges and practical decision making (Figure 1).

This process has proven to be effective and helpful in making the students feel safe, capable, encouraged and embraced. A useful and encouraging statement that I used during the meetings with my students and a reminder for myself when our work energies dropped, intimidated with doubt and a sense of vanity was: “An abnormal reaction to an abnormal situation is normal behavior” [27] (p. 32). I would recommend that mentors and supervisors at academic institutions provide their students and staff with such safe spaces to contain all the stressors conveyed, in order to enable productivity, growth and empowerment when conducting research in a Shared Traumatic Pandemic Reality.

## 5. Conclusions

COVID-19 has imposed many challenges on researchers in conducting an international study on refugees and aid workers’ circumstances in Lebanon. Such topics are rarely investigated and scarcely funded in academic studies, regardless of a pandemic. During the pandemic, many studies have utilized secondhand data found in internet resources due to the limited access to vulnerable populations in the field. However, our team has insisted on facing such challenges and conducted this firsthand study despite COVID-19 limitations and other obstacles faced in “normal” times. To conclude, we have set forth recommendations to the academic community and the humanitarian sector pertaining to conducting an international study and providing services to vulnerable populations in conflict affected zones during COVID-19, respectively, inspired by the holistic approach of humanistic psychology. Our recommendations hope to assist other researchers who undertake uncommon, underrepresented, and underfunded research journeys in times of disasters, beyond COVID-19. First, we recommend that researchers examine the Shared Traumatic Pandemic Reality as encompassing multiple impacts on both participants and researchers. During our study, quadrilateral impacts of COVID-19 were observed on four actors: refugees, aid workers, student researchers and a mentor. The pandemic has affected and changed the life and work circumstances of all mentioned actors, combining an additional layer of secondary traumatization to the already traumatizing work-related materials [21]. Second, conducting in-person data collection during a pandemic would place both participants and researchers at a potential risk of infection. Therefore, adjusting and compromising the study’s goals, design and methodology to address the new subjective conditions of all actors involved in the process are crucial protective measures. Flexibility and authentic communication are key requirements from researchers and mentors to overcome obstacles, such as adjustment of research questions and collaboration with agencies. Researchers need to take into consideration that the findings of their study might be dictated by participants’ personal experiences affected by a pandemic. These may encompass challenges and stressors, as well as new paths of making meaning, growth and development [43]. Third, even though COVID-19 disrupted the speedy processes and productivity of all professions [13], researchers may take advantage of remote studies, since they were found to be cost-effective [31]. Remote research spares the time of travel, accommodation, culture shock and academic institutional reimbursement procedures. Researchers may utilize the time productively in data analysis to be delivered to collaborative agencies in a faster manner. “The methodology should inherently be aimed at improving the situation for those affected by it” [44] (p. 716). Thus, productivity may assist agencies and policy makers in incorporating the research recommendations to the benefit of their beneficiaries and staff. Fourth, being exposed to the complex traumatic pandemic experience, which includes fear, panic, uncertainty, emptiness, economic instability and helplessness, may hold some solace as a shared global human experience [13]. However, at the same time, it may also hold a dehumanizing experience due to the forced quarantine, social distancing and isolation, which can be devastating if not addressed in humane policies. Therefore, we recommend addressing the basic needs of all actors, especially the physiological and safety needs, as well as the need for love and belonging [43]. By providing emotional support during COVID-19, not only to beneficiaries, the refugees in our study, but also to the aid workers who assist them, researchers involved in data collection and mentors who support their research teams may increase the sense of belongingness. Support groups and training on balancing work and work-from-home, remote work and parenting, and precautionary measures during work, are recommended in enhancing the sense of belonging, safety and productivity [45].

Additionally, refugees, aid workers and research teams may face similar challenges during COVID-19, manifested in new work stressors, threatened health, family dynamics, unemployment of family members and other economic obstacles [45,46,47]. Therefore, we also recommend addressing refugees, aid workers, and research teams’ basic needs in providing them with hygiene kits, food pantries or grocery bags, children’s school supplies and so on. Due to COVID-19′s economic impact and the high unemployment rate globally, we recommend continuing the cash assistance for refugees and paid salaries for aid workers and researchers, despite the changes in eligibility criteria, or working hours and productivity, respectively. These recommendations would require flexibility from donors in the terms and standards of the funds provided to academic institutions and relief agencies, so that they can adequately support, embolden capacities, enable a sense of dignity, security and stable functioning of their beneficiaries, staff and researchers.

COVID-19 impacted humanity globally, regardless of citizenship, culture, race, gender, ethnic group, sexual orientation, or any other affiliation, which resulted at times in feelings of despair, meaninglessness, loneliness and a violation of profound aspects of life, such as questioning the purpose in living [48]. Still the impacts of COVID-19 were disproportionately more taxing among sexual minorities, BIPOC and women in general in academia [45], underserved and marginalized populations belonging to a low socio-economic stratum [47], as well as minorities and refugee populations in the Middle East [21,22]. It is thus crucial to familiarize all actors with the term “monoanthropism” or shared humanity [27], to offer them some hope and strength, since they are not alone in the current situation, and to remind them that what they do is meaningful, under all circumstances, including at times of inevitable suffering and torment. Such a reminder will encourage them to make an opportunity out of a challenge and to gain resilience and growth in the face of adversity [48].

This study was an important opportunity to conduct research during unprecedented circumstances, but the recommendations outlined are not only limited to research conducted during COVID-19. The recommendations provided are based on adjusting to the complicated lives of humans and the considerations made will prove valuable to every research study conducted in less-than-ideal circumstances, especially during precarious times of global turmoil. Pandemics unfortunately are persistent and not easily demolished, with the worsening conditions of climate change, spreading of zoonotic diseases facilitated by global trade, as well as the increased displacement of diverse populations worldwide, making coping for professionals even more traumatizing and complex. Thus, it is essential to learn from our experience during COVID-19 in outlining what to expect in such circumstances and tailoring new policies, so that the academic community and humanitarian sector are better prepared when faced with future epidemics and other globally impacting situations.

## Data Availability

Not applicable.
